# Evaluation of the Protective Efficacy of Poly I:C as an Adjuvant for H9N2 Subtype Avian Influenza Inactivated Vaccine and Its Mechanism of Action in Ducks

**DOI:** 10.1371/journal.pone.0170681

**Published:** 2017-01-30

**Authors:** Aiguo Zhang, Hanzhang Lai, Jiahua Xu, Wenke Huang, Yufu Liu, Dawei Zhao, Ruiai Chen

**Affiliations:** 1 College of Veterinary Medicine, South China Agricultural University, Tianhe District, Guangzhou, Guangdong Province, China; 2 Guangdong Enterprise Key Laboratory of Biotechnology R&D of Veterinary Biologics, Guangdong, Zhaoqing, Guangdong Province, China; Sun Yat-Sen University, CHINA

## Abstract

Current commercial H9 avian influenza vaccines cannot provide satisfactory protective immunity against antigenic variant influenza viruses in ducks. Poly I:C, when used as an adjuvant, improves humoral and cellular immunity in many animals but has not been tested in ducks. In this study, we investigated the protective efficacy of Poly I:C as an adjuvant for an inactivated H9N2 Avian influenza vaccine in ducks. We found that an H9N2 vaccine administered with poly I:C (H9-PIC vaccine) induced a significantly more rapid response with higher anti-influenza antibody titers than those of the vaccine alone (H9 vaccine). Moreover, virus shedding was reduced in ducks immunized with the H9-PIC vaccine after challenge with an H9 subtype antigenic variant viruses. IFN-α, IFN-γ, IL-6 and MHC-II mRNA levels were all elevated in ducks receiving the H9-PIC vaccine. In addition, lower expression level of MHC-I may be a reason for inefficient protective ability against heterologous influenza viruses in H9-PIC vaccination of ducks. In conclusion, poly I:C adjuvant enhanced both humoral and cellular immune responses in ducks induced by immunization of inactivated H9N2 vaccine.

## Introduction

Avian influenza viruses (AIV) have a worldwide distribution in a variety of animals including humans, pigs, wild birds and domestic poultry [1, 2]. This group of viruses is responsible for major economic losses to the poultry industry and also responsible for influenza infection in humans [3]. Ducks are the principal natural reservoir for the H9N2 strain of AIV. Interestingly, this antigenic subtype contributed genetic material to the zoonotic H7N9 strain that was responsible for a recent outbreak. Genetic exchange between subtypes plays an important role in AIV dissemination and evolution [1, 2].

Ducks infected with lesser pathogenic H9N2 AIV subtype are generally overlooked due to the lack of clinical symptoms. However, H9N2 can survive for long periods as a persistent infection and be excreted at high concentrations in feces [4, 5]. Vaccination has been proven effective in protecting chickens against AIV and is being used in several countries to control the disease [6]. However, H9N2 underwent significant antigenic drift in China and escaped from protection of the present vaccine [7]. This presents a problem for a vaccination regimen. For instance, Muscovy ducks have a shorter life span than chickens and require a more rapid and efficient immune response after vaccination. The ideal situation would be to shorten the immunization blank period and reach a protective antibody titer more quickly. However, ducks receiving an inactivated vaccine had lower and non-protective antibody titers than chickens. Vaccination also did not effectively eliminate virus shedding into the environment [8–12]. Therefore, we sought to improve serum antibody titers and prevent viral shedding of H9N2 in ducks.

Polyinosinic:polycytidylic acid (poly I:C) is a synthetic double-stranded RNA that can stimulate Toll-like receptor 3 (TLR3) and act as an antiviral agent [13, 14]. Poly I:C is also a safe and effective adjuvant for improving humoral and cellular immunity in animals [15–17]. When poly I:C was used as an adjuvant for the influenza vaccine, it increased AIV immunity and significantly reduced virus shedding in chickens and mice [18, 19]. However, tests such as these are absent for ducks.

Therefore, this study was conducted to evaluate the adjuvant potential of poly I:C for inactivated influenza vaccine (H9N2) in ducks using intramuscular injection. The results were evaluated against heterologous and H9N2 antigenic variants including measuring steady-state mRNA levels of immune response genes induced by poly I:C in duck spleens.

## Materials and Methods

### Ethics statement

All animal experiments were carried out in strict accordance with the guidance of both of the Centers for Disease Control and Prevention’s Institutional Animal Care and Use Committee and the Association for Assessment and Accreditation of Laboratory Animal Care International. The protocol was approved by the Committee on the Ethics of Animal Experiments of Animal Biosafety Level 3 Committee of South China Agricultural University. All ducks of the study have no incidence of animal death or unexpected occurrence was recorded in the course of the experiment. The surgery and euthanasia was performed under anesthesia with sodium pentobarbital solution (100mg/kg body weight) via intravenous route to minimize suffering.

### Viruses and ducks

Three H9N2 viruses isolates, A/Chicken/Jiangsu/SIC12/2013(SIC12/13) (HAclade:4.2.5), A/Chicken/Fujian/SIC9/2013 (SIC9/13) (HA clade: 4.2.5) and A/Chicken/Guangdong/SIC10/2013 (SIC10/13)(HA clade: 4.2.6), were used in this study [20]. Viral stocks were obtained from the Guangdong Enterprise Key Laboratory of Biotechnology R&D of Veterinary Biologics. Virus was propagated in bulk by inoculating into 9–11 day old embryos of specific pathogen free (SPF) chicken eggs. Virus titers were measured following the method of Reed & Muench as described previously [21, 22]. The white Muscovy ducks used in this study were confirmed to be free of any AIV infection [21, 22] and duck groups were housed separately.

### Vaccine preparation

The vaccine was prepared as water in oil emulsion according to a previously described method [23]. The A/Chicken/Jiangsu/SIC12/2013 (H9N2) strain was selected for use as an inactivated vaccine because it is closely related genetically and antigenically to the current endemic H9N2 viruses [20]. Poly I:C (Guangdong Enterprise Key Laboratory of Biotechnology R&D of Veterinary Biologics) was dissolved in sterile PBS for use. Briefly, the H9N2 vaccine candidate virus (50% egg infective dose; EID_50_ at 10^8^/0.1 mL) was propagated in 10-day-old embryonated SPF chicken eggs. The infectious allantoic fluid was inactivated with formalin (final concentration at 0.02%) for 72 h at 37°C as reported previously [24]. Complete inactivation of the virus was confirmed through repeated passages in 10-day-old embryonated SPF chicken eggs and Madin-Darby canine kidney (MDCK) cells as previously described [24]. The inactivated viral antigen was suspended in Tween-80 and poly I:C or PBS. Marcol 52 mineral oil and Span-80 oils constituted the oil phase. One volume of aqueous phase adjuvant was mixed with two volumes of oil phase adjuvant. The formulation of inactivated vaccine contained ≥ 3 × 10^8^ EID_50_ of H9N2 AIV with or without 100 μg poly I:C in a 1 mL volume. The vaccine containing poly I:C was named as H9-PIC vaccine and the control, H9 vaccine.

### Immunization schedule, collection and testing of samples

One hundred and fifty ducks of fourteen-day-old commercial influenza antibody-negative were randomly divided into 3 groups of 50 birds each. Group A: PBS (control group), Group B: H9N2 inactivated vaccine (H9 vaccine group) and Group C: combination of inactivated H9N2 vaccine and Poly I:C (H9-PIC vaccine group). Vaccine or PBS was administered as a single 300 μL intramuscular injection in the thigh muscle at 14 days of age. Blood samples were collected from the medial metatarsal vein (n = 20/per group) on days 7, 14, 21 and 28 post-vaccination (dpv). Serum was collected after a 8,000 ×*g* centrifugation for 2 min at room temperature, and stored at −25°C for hemagglutination inhibition (HI) assays. At the same time (except for 21 dpv), spleen tissues (n = 5/per group) were collected and stored in liquid nitrogen for cytokine mRNA determinations using real-time quantitative PCR (RT-qPCR). All experiments conformed to the Regulation of Animal Experimentation of Guangdong province, China. Serum antibody levels were measured by HI assays performed using a World Health Organization protocol [24]. SIC12/13 (H9N2), SIC9/13 (H9N2) and SIC10/13 (H9N2) were used for HI antigens. HI titers were reported as log2 values.

Total RNA was extracted from selected spleen tissues by TaKaRa MiniBEST Viral RNA/DNA Extraction Kit Ver.5.0 (TaKaRa, Japan) according to the manufacturer’s instructions. Moreover, RNA was quantified by Ultrospec 2000 mass spectrophotometer (Pharmacia Biotech, Uppsala, Sweden).

Approximately 1μg of RNA from each spleen sample was reversely transcribed to cDNA using PrimeScript^™^II1st Strand cDNA Synthesis Kit (TaKaRa, Japan) according to the manufacturer’s protocol [25]. RT-qPCR was performed using a Rotor Gene Q real-time detection system (QIAGEN GmbH, Manheim, Germany). The reaction mixture contained a volume of 20 μL with 10 μL of SYBR Premix Ex Taq^™^ II (Tli RNaseH Plus; TaKaRa, Japan), 2 μL of cDNA and 0.4 mM primers. In these experiments, duck β-Actin was used as the internal control. The amplification program was 95°C for 30 s, followed by 40 cycles with 5 s at 95°C for denaturation, 30 s at 55°C for annealing, and 30 s at 72°C for extension. After the amplification phase, melting curves were routinely performed to confirm the presence of a single PCR product. To validate assays, each sample was tested in triplicate. PCR primers for IFN-alpha (IFN-α) and CD8α were as described previously [26, 27]. Interleukin 2 (IL-2), IL-6, interferon-gamma (IFN-γ), β-Actin, CD4, major histocompatibility complex (MHC) Class I (MHC-I) and II (MHC-II) were designed using Primer 3 software based on published sequences. Primer sequences are summarized in Table 1.

**Table 1:** Primers used in the quantitative real time PCR.

Target gene	Forward sequence (5’-3’)	Reverse sequence (5’-3’)	GenBank no.
IL-2	GACTACAGCTTATGGAGCACCTCT	ACTCCTTTGTGTCATTTGGTGTGT	AF294323
IL-6	CCCAGAAATCCCTCCTCACA	CAAATAGCGAACAGCCCTCAC	JQ728554.1
IFN-α	TCCTCCAACACCTCTTCGAC	GGGCTGTAGGTGTGGTTCTG	DQ861429
IFN-γ	TCAGAGACCTCGTGGAACTGTC	ACTGGCTCCTTTTCCTTTTGG	AJ012254
CD4	GTCCCATCCCACCTAATGTCC	CTCCACCTTTGTTTCACTCTGTTTT	NM001310403.1
CD8α	GAAGTCCTTCAAGGCAGAG	AGACGTCCCTCTTGGTGAC	JX051841
MHC-I	GAAGAGCAAGCAGGGGAAGA	TGAAGCAGAGCGGTTAGACAC	AB115246.1
MHC-II	CTCGAGGTCATGATCAGCAA	TGTAAACGTCTCCCCTTTGG	AY905540.1
β-Actin	TGATGGACTCTGGTGATGGTG	ATTTCTCTCTCGGCTGTGGTG	EF667345.1

### Immune protection experiments

Ducks of the H9, H9-PIC and control vaccine groups were randomly divided into 3 groups of 10 birds each at 28 dpv. The ducks were challenged by an intranasal inoculation using with 5×10^7^at EID_50_ of one of three H9N2 influenza viruses: (SIC12/13, SIC9/13 or SIC10/13) in a 300 μL volume (Table 2). Birds were observed daily for clinical symptoms. Oropharyngeal and cloacal swabs, and sera were collected on days 3, 5, and 7-post challenge for virus isolation and HI assays to estimate the effects of immune protection. Briefly, the swabs were washed and the solutions were serially diluted and inoculated into SPF chicken eggembryos of 10day old for 72 h. The virus titers in the allantoic fluids were measured by HA assay. If the final HA titer was greater than or equal to 2 log2, we considered the duck positive for virus. A detailed protocol previously described was followed [28].

**Table 2:** Recovery of live virus from challenged ducks (positive/total).

Vaccine	Challenge virus	Number of ducks shedding H9 virus/total number		
		3 dpc	5 dpc	7 dpc
H9-PIC vaccine	SIC12/13	0/10^E^	0/10^E^	0/10^e^
H9 vaccine	SIC12/13	2/10 ^E^	1/10^e^	0/10^e^
Control	SIC12/13	10/10	7/10	5/10
H9-PIC vaccine	SIC9/13	5/10^e^	3/10	2/10
H9 vaccine	SIC9/13	8/10	7/10	5/10
Control	SIC9/13	10/10	7/10	6/10
H9-PIC vaccine	SIC10/13	4/10	3/10	0/10
H9 vaccine	SIC10/13	6/10	4/10	2/10
Control	SIC10/13	9/10	5/10	3/10

Note: Ducks were challenged at 28 days post-vaccination. Mixtures of oropharyngeal and cloacal swabs were collected at 3-, 5- and 7-day post challenge as samples for virus isolation. H9 vaccine: inactivated H9N2 avian influenza vaccine; H9-PIC vaccine: inactivated H9N2 avian influenza vaccine containing Poly I:C adjuvant; Control: negative control group. E/e: Different superscripts indicate statistically significant difference for number of positive ducks compared to control group;

^E^: indicate P < 0.01;

^e^: indicate P< 0.05.

### Statistical analysis

Each duck cDNA sample was tested in triplicate. Target gene expression was normalized to a constitutively expressed endogenous control gene (duck β-Actin), and analyzed via the comparative threshold cycle method (2^-ΔΔCT^) method. Statistical analysis was performed used using GraphPad Prism 6 software (GraphPad Software, Inc., San Diego, CA, USA) [29]. Differences in the expression level of immune-related genes and HI levels among ducks vaccinated H9 vaccine, H9-PIC vaccine and PBS were performed using two-way ANOVA followed by a Bonferroni’s test. Number of ducks shedding virus were tested for statistical significance using Fisher’s exact test. Data are shown graphically as the geometric mean of the fold change plus the standard error of the mean (SEM). The results were considered statistically significant when *P*-values were <0.05 [28, 29].

## Results

### Serum antibody responses to vaccination

**Fig 1:**
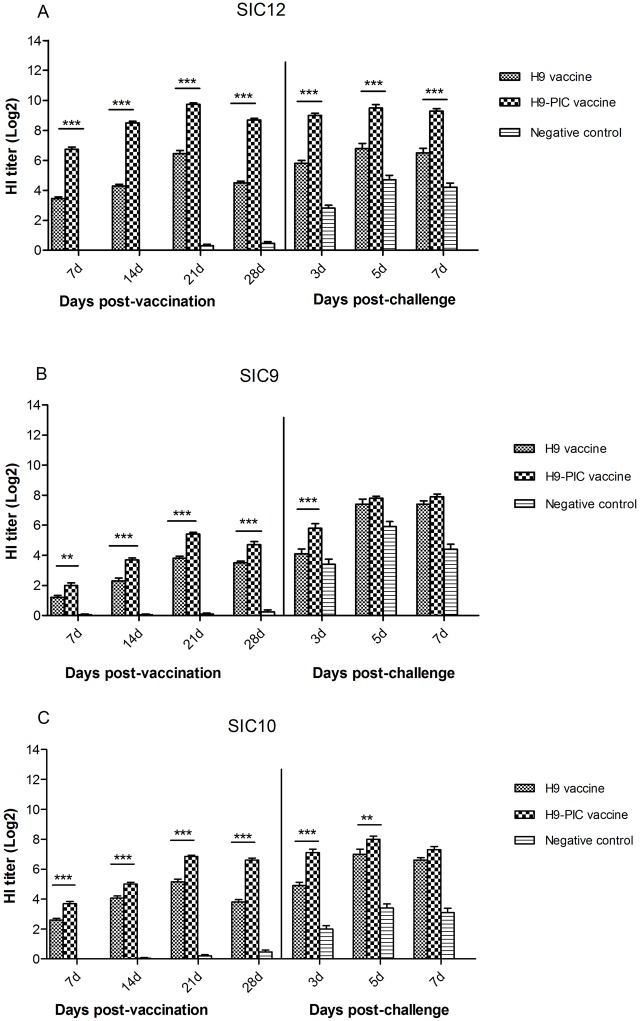
Serum hemagglutination inhibition (HI) levels in vaccinated-challenged ducks. Serum samples (20 ducks per group) were collected at 7-, 14-, 21- and 28-day post-vaccination, and at 3-, 5- and 7-day post-challenge. Antibody titers were determined using the HI assay with 4 HA units of the SIC12/13, SIC9/13 and SIC10/13 avian influenza virus strain, respectively. The HI titer is expressed as the log2 form.Bars indicate mean ± SEM of a representative experiment. (*P<0.05, **P<0.01, ***P<0.001). Experimental groups are as in Table 2.

Serological responses to vaccination and viruses challenge were assessed by HI assays. Before challenge, both of H9-PIC and H9 vaccine groups developed a high HI titers (≥4 log2) when using the vaccine virus antigen (SIC12/13) in the test. The mean HI titer of H9-PIC in ducks of vaccine group increased rapidly and followed amonophasic pattern with a maximum at 10 log2 at 21 dpv. However, the H9 vaccine group developed HI antibodies slowly and peak HI titers were 6.5 log2 at 21 dpv. The value at 28 dpv (4.5 log2) was near the protective threshold for influenza (Fig 1A). Also a high HI cross reactivity (≥4 log2) were observed in H9-PIC and H9 vaccine group after 14 and 21dpv when using the challenge virus antigen (SIC10/13) in the test, respectively (Fig 1C). When using the SIC9/13 virus antigen in the test, lower levels of HI cross reactivity (<4 log2) was observed in H9 vaccine group during the 28 days monitoring period; however, ducks of H9-PIC vaccine group developed a high HI cross reactivity (≥4 log2) after 21 dpv (Fig 1B). In all, the HI titers of ducks immunized with H9-PIC vaccine showed an insignificant elevation compared with those of the ducks that received only H9 vaccine (Fig 1). After challenge, all of the control groups duck had positive titers when using the challenge viruses in the HI tests (>3log2) indicating exposure and replication of the challenge viruses. Also, high post-challenge titers were observed in the H9 vaccine groups ducks when challenge by the viruses (Fig 1). However, the HI titers of H9-PIC vaccine group had not significant elevation compare to the 28 dpv when challenged by SIC12/13 virus during the experiment (Fig 1A).

### Expression of immune-related genes in vaccinated ducks

**Fig 2:**
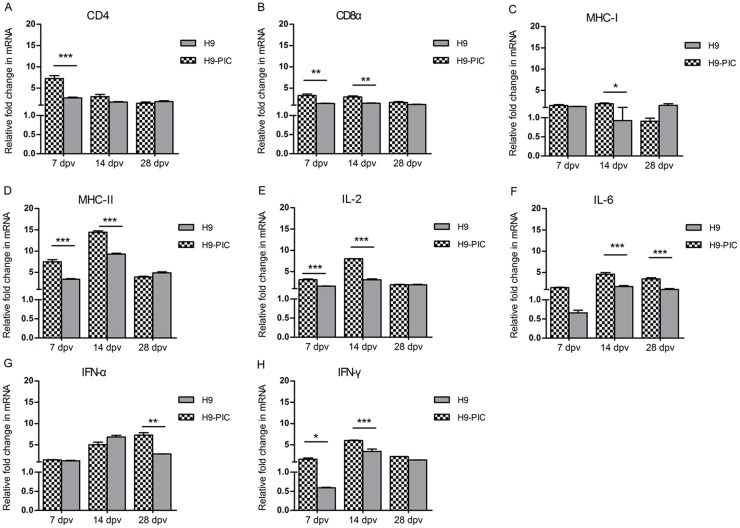
The mRNA expression levels of immune genes in duck spleens. Spleens of ducks immunized with H9 vaccine, H9-PIC vaccine and PBS were harvested for RNA isolation at 7-, 14-, and 28-day post-vaccination. The mRNA levels for CD4 (A), CD8α (B), MHC-I (C), MHC-II (D), IL-2 (E), IL-6 (F), IFN-α (G) and IFN-γ (H) were evaluated by real-time PCR. Expression of target genes was normalized to those of duck β-Actin. The relative fold change was calculated using PBS injected ducks as control. Bars indicate mean ± SEM of a representative experiment. (*P<0.05, **P<0.01, ***P<0.001). Experimental groups are as in Table 2.

To identify potential immunological correlates of protection, gene expression in the spleen was examined on days 7, 14 and 28 dpv using real-time PCR. When the H9-PIC and the H9 vaccine groups were compared, the mRNA expression levels of CD4 *(P* < 0.001) and CD8α (*P* < 0.01) were significantly increased at 7 dpv in H9-PIC, and showed no differences at 28 dpv. In addition, CD8α mRNA levels were significantly increased (*P<*0.01) in the H9-PIC group but CD4 levels were not different at 14 dpv (Fig 2A and 2B). When we measured MHC expression, class II levels were up regulated at 7 and 14 dpv (*P*<0.001) compared to the H9 vaccine group. Class I levels remained constant throughout the experiment (Fig 2C and 2D). Expression levels of both IL-2 and IL-6 increased significantly in a biphasic pattern in the experimental group, but the maxima appeared at different days between the two. There was also a significant elevation of IFN- α mRNA levels in the H9-PIC vaccine group towards the end of the experiment, compared to the H9 vaccine group (Fig 2G). Conversely, IFN-γ mRNA levels were significantly elevated at 7 dpv (P< 0.001) with a maxima at 14 dpv (P< 0.05). There were no differences between the H9-PIC and control group at the final stages of the experiment (Fig 2H).

### Protective efficacy and viral shedding after viral challenge

There were no deaths in any of the animals during the 7-day observation period after virus challenge. A series of clinical symptoms were observed in all control groups and partly in the H9-PIC and H9 vaccine groups. These symptoms included listlessness, anorexia and nasal discharge at 3 dpc. Oropharyngeal and cloacal swab samples were collected at 3, 5 and 7 dpc for virus isolation. No live virus was isolated from the ducks challenged by A/Chicken/Jiangsu/SIC12/2013 (H9N2) during the 7-dpc observation period in the H9-PIC vaccine group. However, two birds in the H9 vaccine group and all birds in control group were positive for virus isolated at 3 dpc. A similar pattern was seen when the A/Chicken/Fujian/SIC9/2013 (H9N2) and the A/Chicken/Guangdong/SIC10/2013 (H9N2) strains were used (Table 2). Interestingly, A/Chicken/Guangdong/SIC10/2013 (H9N2) could not be isolated in the H9-PIC vaccine group at 7dpc. The control group had two positives during this period (Table 2).

## Discussion

Domestic ducks are the natural reservoir of H9N2 that not only leads to huge economic losses in the poultry industry, but also threatens public health. The current H9N2 subtype inactivated vaccine only induces a low protective antibody titer in ducks. It does not effectively eliminate virus shedding in the field [8–12]. When poly I:C was used as adjuvant in previous studies, it enhanced both humoral and cellular immune responses [15, 30, 31]. Its mechanism of action is based upon type I IFN production that enhances dendritic cell maturation and B cell activation. This ultimately leads to induction of potent CD4^+^ T cell and humoral immune responses [11, 23]. Type I IFN is critical for cross presentation of protein antigens to generate CD8^+^ T cell responses in mice [13]. However, this type of data is lacking for duck infections, especially concerning the immune responses in immune organs.

In our study, after vaccination, the HI assay showed that antibody levels of the H9-PIC ducks increased rapidly and reached 6.8 log2 at 7 dpv compared with 3.5 log2 of the H9 control group when using the vaccine virus antigen (SIC12/13) in the test. Also, the H9-PIC group ducks maintained high-titer antibody levels from 7 dpv to 28 dpv when compared to the H9 vaccine group. Similar results have been observed in mice and pigs [15, 31]. In addition, the levels of cross-reactive HI antibodies were examined in this study. The results showed that higher HI titers in the H9-PIC group as compared to H9 group at the 14-, 21- and 28 dpv, which may be involved in poly I:C as adjuvant improved serum HI antibody levels and inducing cross-protective immunity against antigenic variant SIC9/13 and SIC10/13 viruses. Altogether, it indicates that poly I:C was successful as an adjuvant and enhanced humoral immune responses in ducks; while previous studies mainly suggested that poly I:C as adjuvant could improve cellular immunity to antiviral infection through the production of interferon [32, 33].

Also, in our study, IFN-γ, MHC-II, CD4 and IL-6 genes were all significantly up-regulated in spleens of the H9-PIC group at 7 and 14 dpv, consistent with previous studies [34]. In addition, poly I:C has been shown to induce significant increases in IL-6 expression in lymph cells [35] and with CD4 levels in mice [13]. Furthermore, during the latter parts of our experimental period, IFN-γ and CD4 were not significantly differential though MHC II and IL6 were significantly up-regulated at 28 dpv in the H9-PIC group. In mammals, CD4 T helper cells play a central role in immune responses [34]. IL-6 is a Th2 cytokine and induces antibody production in B cells, and promotes T cell activation and differentiation [36]. Therefore, we suggest that in our experiments, CD4 and IL-6 also contributed to enhanced antibody levels induced by poly I: C in ducks. In particular, IL-6 may play an important role in maintaining high antibody titers at later times in the immune response.

The H9N2 virus has low pathogenicity to poultry and could not induce obvious signs in ducks. However, H9N2 AIVs were reported to serve as an progenitor for novel human avian influenza viruses including H5N1, H7N9 and H10N8. Hence, H9N2 poses a serious threat to public health [4, 5, 36]. Although existing vaccines reduce the incidence of morbidity and mortality in infected poultry, they do not completely prevent infection. They are, therefore, not capable of preventing shedding of the influenza virus into the environment [7–12]. When poly I:C is used as an adjuvant, it broadens protection against H9 heterologous virus challenge and reduces AIV replication and shedding in chickens [37]. In this study, ducks receiving the H9-PIC vaccine were fully protected against homologous virus challenge. In contrast, virus shedding in ducks of the H9 commercial vaccine group was greater compared to the H9-PIC group after the antigenic variant H9N2 was used for challenge. This result indicates that poly I:C when used as an adjuvant could enhance cell-mediated immune responses and broaden protection against H9 heterologous virus challenge in ducks. Similar results have been found in mice and pigs [19, 31]. Th1 cells induce IL-2, IFN-α and IFN-γ cytokines which promote T-cell cytotoxicity. In our study, expression of these three genes were increased to different levels in spleens of the H9-PIC group compared to H9 vaccine group. CD8+ cells are cytotoxic cells that recognize and kill host cells infected with intracellular microbes. Compared to the H9 vaccine group, the mRNA expression level of CD8α was significantly increased at 7 dpv (*P*<0.01) in the spleens of H9-PIC vaccine group. This means that poly I:C as adjuvant showed a positive effect on duck cellular immunity.

IFN-α has been shown to protect ferrets and chickens against influenza viruses [38, 39]. After poly I:C stimulation *in vitro*, peak IFN-α levels are reached at 4 h and down-regulated by 48 h [40]. However, in our study, IFN-α expression showed the opposite results; there was up-regulation at 28 dpv, but not at earlier times. We can offer no explanation at present and this situation needs further analysis. Poly I:C also enhances MHC I expression in mouse cells *in vitro* [41]. However, there were no obvious increases in MHC I expression in the spleens of the H9-PIC group in the present study. This is an interesting finding because MHC class I molecules present endogenously derived peptides to CD8^+^ T-cells, which play an important role in cytotoxic T-lymphocyte responses. This is a possible explanation for the inability of the H9-PIC vaccine to provide better protection from heterologous influenza virus challenge as has been seen in previous studies, even though the factors of such as vaccination strategy, the duck species and the virulence of the challenge virus used also should be considered [18, 19, 31].

## Conclusions

This study demonstrated that H9-PIC vaccine more rapidly induced elevated antibody levels and reduced viral shedding against the heterogeneous antigenic variant H9N2 influenza in ducks. Poly I:C adjuvant could significantly improve the efficacy of an inactivated H9 subtype AI vaccine in both humoral and cellular immunity in ducks, suggesting that Poly I:C adjuvant for vaccine could be used to prevent and control H9N2 influenza in ducks.

## Supporting Information

S1 File The file contains the original data of HI assay used in this study. (XLSX)

S2 File The file contains the original data of RT-qPCR used in this study. (XLSX)
